# Evaluation of a joint Bioinformatics and Medical Informatics international course in Peru

**DOI:** 10.1186/1472-6920-8-1

**Published:** 2008-01-14

**Authors:** Walter H Curioso, Jacquelyn R Hansen, Arturo Centurion-Lara, Patricia J Garcia, Fredric M Wolf, Sherrilynne Fuller, King K Holmes, Ann Marie Kimball

**Affiliations:** 1 School of Public Health and Administration. Universidad Peruana Cayetano Heredia, Lima, Peru; 2 School of Medicine. Universidad Peruana Cayetano Heredia, Lima, Peru; 3 University of Washington, Seattle, Washington, USA

## Abstract

**Background:**

New technologies that emerge at the interface of computational and biomedical science could drive new advances in global health, therefore more training in technology is needed among health care workers. To assess the potential for informatics training using an approach designed to foster interaction at this interface, the University of Washington and the Universidad Peruana Cayetano Heredia developed and assessed a one-week course that included a new Bioinformatics (BIO) track along with an established Medical/Public Health Informatics track (MI) for participants in Peru.

**Methods:**

We assessed the background of the participants, and measured the knowledge gained by track-specific (MI or BIO) 30-minute pre- and post-tests. Participants' attitudes were evaluated both by daily evaluations and by an end-course evaluation.

**Results:**

Forty-three participants enrolled in the course – 20 in the MI track and 23 in the BIO track. Of 20 questions, the mean % score for the MI track increased from 49.7 pre-test (standard deviation or SD = 17.0) to 59.7 (SD = 15.2) for the post-test (*P *= 0.002, n = 18). The BIO track mean score increased from 33.6 pre-test to 51.2 post-test (*P *< 0.001, n = 21). Most comments (76%) about any aspect of the course were positive. The main perceived strength of the course was the quality of the speakers, and the main perceived weakness was the short duration of the course. Overall, the course acceptability was very good to excellent with a rating of 4.1 (scale 1–5), and the usefulness of the course was rated as very good. Most participants (62.9%) expressed a positive opinion about having had the BIO and MI tracks come together for some of the lectures.

**Conclusion:**

Pre- and post-test results and the positive evaluations by the participants indicate that this first joint Bioinformatics and Medical/Public Health Informatics (MI and BIO) course was a success.

## Background

In the last decade, the field of health informatics has experienced extraordinary growth, and the demand for health professionals with skills in this area is growing [1].

Advances in global health may be increasingly driven by new technologies that emerge at the interface of computational and biomedical science [2]. Bioinformatics initially was synonymous with DNA and protein sequence data management and analysis, but now it has rapidly expanded with the rapid progress in full genome sequencing, functional genomics, pharmacogenomics, proteomics, metabolomics research, and biopathway modeling [3]. These approaches are providing great insights into many questions about biology, evolution, ecology, and public health. Despite the fact that a large number of genome sequences are from pathogens that represent a significant burden for global health, involvement of researchers from developing countries in genome projects and timely development of bioinformatics capacity in these regions is rather limited [4]. In the global context, bioinformatics is crucial to the future of biotechnology for developing countries in promoting more effective and efficient methods in understanding the processes of disease and health, and in developing new or better drugs.

In developed countries, medical informatics have been taught over the last 20–25 years, and their successes and setbacks have been well-documented, providing good models for future course development [5-11]. In developing countries, however, training of health professionals in informatics remains one of the biggest challenges. Inadequate education in informatics skills is a constraint among medical students, doctors, nurses, and many other health care professionals who have varying levels of computer competence [12-14]. In a study conducted by Horna et al., 40% of a sample of medical students in Peru' reported lack of proficiency on the use of Internet [15]. Similarly, in 2003, Samuel et al. reported that only 52% of medical students in Tanzania felt that they understood the basic terminology and concepts of computing. In Nigeria, Ajuwon reported that only 42.6% of a sample composed of medical and nursing students could use a computer [13]. Another study conducted in Nigeria reported that 79% of medical and dental students had little or no computer skills [16].

Some training experiences in health informatics have been described from Latin-America, Africa, and Asia [16-21], including on-line training [20], on-site and online education [22], and even formal training in some academic curricula [23], but much more is needed. There is a need for collaborative alliances or partnerships to enable provision of global health informatics education [24].

Peru, a middle income Latin American country, still faces significant challenges to improving health for its people. Much like other Latin American countries, Peru has infectious disease epidemics such as multi-drug resistant tuberculosis and malaria.

In Peru, formal master's or doctoral-level programs in health informatics among universities are beginning, and there is a lack of other health informatics training programs, such as short courses, certificates programs, and diplomas. But Peru does offer the AMAUTA (Quechua word for master) Global Informatics Research and Training Program for researchers in the region. AMAUTA is a collaborative partnership between the Universidad Peruana Cayetano Heredia (UPCH) in Lima and the University of Washington in Seattle. The program is funded by the National Institutes of Health (NIH) Fogarty International Center and the National Library of Medicine.

The University of Washington (UW) and its International Training in Health Informatics Program (ITHIP) has twice offered a two-week intensive short course in Lima (2000 and 2001) in collaboration with two schools of medicine [18]. Those courses provided an introduction to medical informatics. The AMAUTA program then organized an advanced course entitled Informatics for Global Health: Advances in Public Health and Genomics, which was held in November 2005. This paper describes the evaluation of the course.

## Methods

### Development of the course

The objectives of the course were as follows: 1) to present the state-of-the-art developments in medical informatics, public health informatics, and bioinformatics; 2) to increase the knowledge of participants in the application of recent information technology tools in medicine, public health, and biomedical investigation in Peru, 3) to enable participants to develop and strengthen collaborative studies both locally and internationally; 4) to present and summarize research projects on medical/public health informatics and bioinformatics being conducted by Peruvian fellows (present and past) of the ITHIP, and, 5) to improve the use of current informatics tools by the participants to form new working hypotheses, and to conduct new collaborative research projects concerning global health.

The course was structured in two parallel tracks: one with a Bioinformatics focus (BIO), and one with a Medical Informatics/Public Health Informatics focus (MI). The Bioinformatics track was added due to the rapidly growing demand for bioinformatics training in Peru. Both tracks had separate workshops and instruction but shared several lectures. Topics were selected by course coordinators and course faculty from UPCH and UW based on feedback received from previous short courses and based on research interests of the course faculty. We solicited topics from Peruvian faculty based on the priorities for institutional and content development. The course was targeted for a wide-range of health-related professions: clinicians, nurses, biologists, bioinformaticians, librarians, physicists, mathematicians, chemists, public health professionals, laboratory investigators, and health science students.

For both the MI track and the computer laboratory sessions, a computer with Internet access was provided to each participant for use during the course.

### Participant selection

Of the more than 90 candidates who expressed interest in the course, 64 submitted applications and 43 were accepted. Applications were submitted online and included a self-reported skills survey to determine computer proficiency. Five faculty members from UPCH selected the participants. The criteria for selection included the participant's affiliation with an academic institution, experience with medical informatics and/or a bioinformatics project, relevant publications, and availability to attend the full course. The participants were almost equally divided between the tracks – 20 for Medical Informatics/Public Health Informatics and 23 for Bioinformatics. Full scholarships were offered for all participants.

### Course Description

The MI track included five topics: Electronic Medical Records, Genomics, Open Source, Organizational Issues in Health Informatics, and Surveillance and Evaluation of Health Informatics Systems. The BIO track included four topics: Computational Biology, Drug Design, Organizational Issues in Health Informatics, and Genomics. The BIO and MI tracks shared two topics in common – Organizational Issues in Health Informatics, and Genomics. A complete list of topics is described below:

• Informatics in public health. Introduction and review^1^

• Fundamentals of surveillance. Symptomatic surveillance^1^

• Use of Web-based systems for management of patients with TB and HIV^1^

• Drug order entry and drug supply management in Peru and Haiti^1^

• Study of the use of PDA's for web-based collection of TB bacteriology results^1^

• Grant writing 101^1^

• Information systems for clinical studies. Web based management systems^1^

• Functional genomics^1^

• Parasite databases^1^

• Open source and free software: Relevance in biomedical research^1^

• Using the Internet and computers as tools for STD/HIV prevention^2^

• Annotation^3^

• Phylogenetics^3^

• Microrrays^3^

• Proteomics^3^

• Information specialists and their role in health informatics^1^

• Information retrieval and virtual libraries^1^

• Drug design^3^

• Use of cell phones in public health surveillance systems^2^

• Metanalysis^2^

• Genomics and syphilis research^3^

• Whole-genome mapping and resequencing^3^

• Promoter arrays and ChIP-on-chip analysis^3^

• Distributed information systems^1^

• Privacy, confidentiality, and security in health informatics^1^

• Organization and management issues in informatics^1^

• Designing and evaluating health information technologies^1^

• Future directions on health informatics^1^

• Computational biology^1^

• Sequence analysis workshop^3^

^1 ^= For both tracts, ^2 ^= Only for Medical Informatics ^3 ^= Only for Bioinformatics

A free public mini-symposium was presented at the beginning of the course. The course was conducted by five faculty members from UPCH, nine from the University of Washington, two from Harvard University, and two from the U.S. National Institutes of Health.

Novel technologies were used during the course. For example, an interactive, wireless, PowerPoint compatible device Audience Response System (TurningPoint^® ^Response Card RF) was used to engage students with questions that allowed them to gain insight from different people in real time. After students had responded to the questions, results were immediately displayed graphically on a PowerPoint slide that indicated the percentages students responded to each of the question alternatives.

During the course, we hosted two free public teleconference sessions: one on meta-analysis led by a speaker at the University of Washington and one on grant writing led by speakers at the U.S. National Institute of Health. For the teleconference on meta-analysis, we used Macromedia Breeze (Adobe Systems, Mountain View, Calif.), which allowed participants to pull up a lecture using both video and audio simultaneously. For the session on grant writing, we presented a PowerPoint presentation using the software program Elluminate Live! (Elluminate, Ft. Lauderdale, Fla.), which allowed simultaneously live audio and sharing a whiteboard for notes.

The course Web site in Spanish was at: http://faculty.washington.edu/wcurioso/curso.htm

## Evaluation

### Knowledge

Students completed 30-minute pre- and post-tests specific for each track (MI and BIO). Each test was paper-based and consisted of 20 multiple-choice questions. The questions were selected from an original pool of 35 questions developed from the subject matter to be covered by the course. Questions on the pre and post-tests [see Additional file 1] were identical; however, the order of presentation of the individual items was randomly changed on the pre-test. The students were not allowed access to the examination outside the testing period to prevent unauthorized distribution of the questions.

### Attitudes

Two systems were used to evaluate participant satisfaction: 1) A daily evaluation of each session based on informative content, usefulness, and presentation techniques, and 2) an end-of-course evaluation that consisted of questions concerning strengths, weaknesses, effectiveness, and general comments about the course.

The daily evaluation, administered at the close of every day, used a Likert scale (rating of 1 to 6 where 1 was very poor and 6 was excellent) for each session with an area to comment on each one. The end-of-course evaluation, administered after the post-test, was composed of four Likert scale questions as well as open-ended questions inviting general thoughts on the course. Other items asked respondents to rate the value of having attended the course and to indicate preferences regarding topics for future courses.

### Follow-up Evaluation

A follow-up, confidential, Web-based survey was sent by e-mail to all participants six months after conclusion of the informatics course to evaluate the impact of the course in their current work.

Included in the survey were questions asking respondents if they were actively involved in health informatics or bioinformatics-related activities (i.e., project, teaching, etc.) at the time of the six-month survey, whether they thought the course had changed their vision of what health informatics is, and whether they used what they learned after the course. Respondents were also asked to rate how useful the informatics course was, and if they were willing to participate in a health-informatics diploma at UPCH. They were also invited to provide additional comments.

### Statistical Analysis

Descriptive statistics were used to summarize the course. Paired *t*-tests and effect size measures were used to assess knowledge improvement for each track. Effect size measures are useful in assessing and interpreting the magnitude of knowledge change, and complement *t*-tests, which only assess the statistical significance of change. We used Cohen's *d *statistic, which was obtained by dividing the difference between the pre- and post-test means by the within-group standard deviation, and provide a standardized measure of change [25]. A Cohen's *d *value of 0.20 indicates a small effect size, 0.50 a medium effect size and 0.80 indicates a large effect size [25]. The test scores from the pre-tests and post-tests were entered into computer files using the social science statistical software (SPSS version 13). Data are presented as mean (standard deviation or SD) scores or numbers (percentages). Paired *t *tests were used to determine whether there had been a statistically significant change between the pre- and post-test scores. For all tests, significance level was α = 0.05.

Qualitative data from the daily evaluation sessions and final group evaluations were analyzed separately. The results from each evaluation provided descriptive information about how participants experienced the course.

## Results

### Participants

Of the 43 participants, 27 (63%) were male. Professional backgrounds of the participants varied between tracks. The 20 Medical Informatics track participants were more diverse: nine were physicians, three informatics engineers, three librarians, one midwife, one nutritionist, one nurse, one public health worker, and one statistician.

Of the 23 participants of the BIO track, 15 were biologists, three pharmacists, two physicists, one chemist, one informatics engineer, and one mathematician. Most of the participants were affiliated with the UPCH; others came from other medical institutions, non-governmental agencies, and the Ministry of Health. Two of the participants were not Peruvians: one was from Colombia, one was from the United States. The rest of the participants were primarily from the Lima metropolitan area.

### Self-Reported Informatics Background and Skills

The baseline self-reported skills survey found that the Medical Informatics group had less skill in speaking English and in using PubMed/MEDLINE. The Bioinformatics group had higher scores in reading English, speaking English, computer skills, and using PubMed/MEDLINE. Overall, 65% self-reported very good proficiency in use of computers, which was helpful for a fuller understanding of this course.

### Knowledge Gains

Eighteen of 20 in the Medical Informatics track and 21 out of 23 in the Bioinformatics track completed both the pre- and post test. Results of performance for 39 health professionals on the test administered at the beginning and close of the course is summarized in Table 1.

**Table 1:** Percentage correct, paired *t*-tests and effect sizes (Cohen's *d*), for 39-health professional's performance on their knowledge test before (pre-test) and after (post-test) the course

**Track**	N*	**# of questions**	**Pretest mean % (SD)**	**Posttest mean % (SD)**	**Within SD**	***t *±**	**Cohen's *d***
BIO	21	20	33.6 (10.4)	51.2 (13.1)	11.8	5.7	1.49
MI	18	20	49.7 (17.0)	59.7 (15.2)	16.1	3.7	0.62

* N represents number of symposium participants who completed both the pre- and posttests.

± All t-tests are significant at *P *< 0.05 (two-tailed tests).

The BIO group showed a greater improvement in overall score. Participants' performance on the knowledge test improved from pre- to post-testing from 33.6% to 51.2% for the BIO group and from 49.7% to 59.7% in the MI group. Individual *t*-tests of these gains for the two tracks were significant, *P *< 0.05 in both tracks.

The MI group had an increase in test scores for every one of the five subject areas, while the BIO group had an increase in three out of four subject areas (Drug Design did not show an increase).

The size of the effect (Cohen's *d*) on knowledge gains indicated that participant's knowledge in the BIO group had a large effect size and the MI group had a medium effect size.

### Attitudes

#### Daily

A total of 96 daily evaluations were completed over the five days of classroom sessions, with 515 written comments registered. Thirty-two topics were covered in the week-long course, 24 of them had ratings between 4 and 4.99, and eight had ratings of 5 or above, showing that the courses had an average rating of good or better. Of the written comments, 391 (76%) were clearly positive with the other 124 (24%) being either negative or with both negative and positive feedback included in the one comment.

#### End of Course

The end-of-course evaluation on the last day included four rating questions using the Likert scale and four open-ended questions. Of the participants, 18 out of 20 in the Medical Informatics track and 22 out of 23 in the Bioinformatics track completed the evaluation. Results are shown in Table 2 for both tracks.

**Table 2:** End-of-course evaluation scores for both tracks

Question	MI Mean (SD)	BIO Mean (SD)
Overall rating of the course 1 = Poor; 2 = Fair; 3 = Good; 4 = Very Good; 5 = Excellent	4.2 (0.62)	4 (0.69)
Amount of information 1 = Too little; 2 = Less than adequate; 3 = Adequate; 4 = More than adequate; 5 = Too much	3.2 (0.51)	3.3 (0.65)
Usefulness of the course 1 = Poor; 2 = Fair; 3 = Good; 4 = Very Good; 5 = Excellent	4.2 (0.62)	3.9 (0.71)
Would you recommend this course to your peers? 1 = Definitely not; 2 = Unlikely; 3 = Likely; 4 = Definitely yes	3.8 (0.43)	3.6 (0.50)

The main perceived strength of the course was the quality of the speakers and the state-of-the-art technology used to present information. The comments were overwhelmingly positive. Said one participant: "It's the best course I have attended to up to this date. Informatics is a science applied to several fields, such as Medicine and Biology, and many people (professionals) don't use it due to a lack of knowledge, but my congratulations to UPCH, which is always ahead."

Students enjoyed the novel technology resources used, such as the interactive audience response system (which provided immediate feedback) and the teleconference sessions. This was cited also as one of the perceived strengths of the course.

The main perceived weakness was the short duration of the course. People wanted more time to interact with their peers. Some of the attendees did not know each other. They wanted more opportunities to interact with informatics technological tools.

Having the joint sessions caused a mixed reaction, generally positive. Among the medical informatics group, only half of the 17 respondents thought that it was an excellent or interesting approach. Said one participant: "It allows having a wider vision of informatics in general." But most people (13 out of 18 respondents) in the bioinformatics group thought that it was a great idea. Said one respondent: "I am a biologist and I did not know that they also used informatics in medicine, and now I know another very important aspect. I learned the value of arranging my data with informatics tools."

### Follow-up Evaluation Results

Of the 43 participants 29 (67%) responded to the six-month follow-up survey. Of those, 23 (79%) were actively involved in health informatics or bioinformatics-related activities (i.e., project, teaching, etc.) at the time they completed the survey. Of those who responded, 24 (83%) thought that the course had changed their vision of what health informatics is. For example, 18 (75%) perceived that the course increased their knowledge in informatics, and 5 (21%) perceived that the course provided participants more informatics skills. Examples were: "The course expanded my horizons and showed clearly the advantages of the use of informatics and telemedicine"; "After the course, I started using bioinformatics tools to compare genomes and bacterial DNA sequences"; "I had a more clear and objective vision on how informatics could be applied in public health such as patient monitoring, databases, etc." Also, 24 (83%) have used what they learned after the course. For example, participants were using bioinformatics tools, improving database searching and designing new drugs with informatics tools.

All the respondents to the follow-up survey rated the usefulness of the course (scale range: 1 = poor – 5 = excellent) as "good," "very good," or "excellent." There was not a statistically significant difference between the mean (SD) of the end-of-the-course evaluation (3.9, 0.65) and the mean (SD) of the six-month follow-up evaluation (3.8, 0.63). This is a positive result in that there was no significant decline over time.

Twenty (69%) respondents to the follow-up survey expressed their willingness to participate in a health-informatics diploma offered by UPCH.

## Discussion

Overall, the course was described by the participants as a positive experience. These comments came from the daily evaluations, and by the individual written comments at the end of the course. Knowledge increased during this course based on the pre- and post-test scores. Gains in knowledge assessed immediately at the end of the course indicated that this is an effective method of information dissemination. The National Institute of Education has issued guidelines asserting that gains as small as 0.33 standard deviation units are indicative of an educationally significant effect [26]. Gains from pre-test knowledge exceed this criterion for the BIO and MI groups, although the BIO group had a larger effect size than the MI group. These results support the hypothesis that these types of courses can be very effective vehicles for translating and transferring current informatics knowledge to health professionals. However, longer-term retention of these knowledge gains were not assessed, which is a limitation of this study. This increase of knowledge is consistent with the success of other short informatics courses in Peru [18,27]. For example, in the course held in 2000 out of 31 questions, the mean % score increased from 53 pre-test to 71 for the post-test. In 2000, the overall acceptance of the course was very good to excellent with a rating of 4.2 (scale 1–5), and the usefulness of the course was rated as very good.

The Bioinformatics track added for the first time to a health informatics course in Peru, was well-received and showed an increase in knowledge among the participants. With the addition of the new track, a new strategy was also introduced. Instead of a two-week course (as the previous two courses), the course was one intensive week with two tracks running simultaneously, mainly because health professionals reported little time for training. This brought mixed reactions and was the leading negative comment in the end-course evaluation. The length of the course needs to be examined more thoroughly in further courses. The American College of Medical Informatics Task Force takes no formal position on optimal length of training for health professionals interested in informatics [28]. The debate is still going on in the education working group in Medical informatics within the American Medical Informatics Association [29], the International Medical Informatics Association [30], and the European Federation for Medical Informatics [31]. Special debate is going on concerning the role and importance of bioinformatics, as one generally important part in the field of health informatics.

Training programs come in many shapes and sizes, and trainees have a wide range of needs. Informatics training for health professionals is a process. Serious training in biomedical informatics, irrespective of application domain, typically requires focused and extended study. It is not possible to become a medical or health informatician, in any sense of the word, through attendance of a lecture series or participation in a series of workshops [28].

The results of the scores from both tracks indicates a lower post-test score than the last informatics course (2001) [27]. This issue could be due to several factors:

1) The complexity of the new topics introduced. The concepts may have been too difficult for participants or not presented clearly.

2) The short duration of the course not allowing enough time to absorb the new information.

3) The level of difficulty of the test. The test may have been too advanced for these participants. Three of these five questions came from the drug design subject area, a subject that should be readdressed.

4) It is possible that some of the questions required more knowledge than was able to be covered in the sessions. Through observation and informal interviews, there did not seem to be adequate time to complete the Bioinformatics test, but this could also be due to the difficulty level as well.

5) Some participants missed sessions, and there was confusion over the translation in both the tests and the sessions. These factors were also mentioned as potential limitations in the 2000 Health Informatics Course [18].

6) Information overload – too much information over a short period of time leading to poor test performance [32].

The predominantly positive scores from the daily evaluations indicate satisfaction among participants with both with the sessions and the speakers. This satisfaction with the speakers is further supported by the fact that having good speakers was the most often mentioned strength of the course. It was beneficial to include speakers from both Peruvian and non-national institutions to give greater expertise and diversity to the course. It was the first time that a mini-symposium was included with former Peruvian scholars trained in health informatics at the University of Washington. This symposium gave the scholars an opportunity to present their work and network for potential research collaborations [33]

The interactive response system popular with participants has been successfully used in other settings such as in a group of medical students at the University of Washington too [34].

The leading weakness cited by participants was the short length of the course. Other related weaknesses mentioned included not enough practice time and too much information (only for BIO). These weaknesses could be improved for the next courses if a longer course is planned for a slower acquisition of skills, and more "hands-on" time is given to practice these skills.

The course was both celebrated for its diversity of topics, yet criticized for some lectures not being appropriate. This result is not surprising when bringing together professionals from a diversity of backgrounds and experiences so that not everyone will be completely satisfied with the content of every session. Nevertheless, the opinions of the joint sessions were on the whole quite positive. Of interest, there was a higher percentage of MI participants that had negative comments concerning the joint sessions than the BIO participants. One possible explanation is that the general informatics related content in the MI part of the course was more general and thus useful to the BIO participants, while the BIO content may have been more specific and perhaps too detailed for the MI participants

The six-month follow-up survey found that the majority of people were actively involved in health informatics or bioinformatics-related activities (i.e., project, teaching, etc.) at the time they completed the survey. In addition, the majority of respondents thought that the course had changed their vision of what health informatics is, and have used what they learned after the course. Some people found new informatics-related jobs after the course. Said one participant: "This course changed my viewpoint and gave me the confidence to search for a job in informatics. Right now, I am a program manager developing a software program targeted to unify health services for people living with HIV/AIDS." After six-months, respondents maintained their perception on rating the usefulness of the course as very good.

### Course strengths

The present course had several strengths:

1) Promotion of interactions and collaborations between speakers and participants to develop potential topics for future research projects.

2) Multidisciplinary topics and faculty from different institutions (UPCH, UW, Harvard, NIH).

3) Generation of interest of bioinformaticians in medical/public health research and vice versa.

### Course limitations

The sample of the participants for the course was non-representative since selection was limited both by class size and by how participants learned about the course. The participants consisted mainly of adult Peruvian professionals interested in health informatics and bioinformatics.

## Conclusion

This course can be seen as a success based on significant improvements in pre- to post-course test scores, and the positive evaluations by the participants in both tracks. The predominantly positive comments also indicate that the new addition of the bioinformatics content, along with the joint lectures involving both tracks, was a success. The quality of speakers, noted to be the main strength, could be attributed to the use of speakers from multiple institutions.

The appropriate length for the course to avoid information overload still needs further definition. Also, more examination of the course design needs to occur to ensure that the topics and content are specific to the participants' interests and appropriate for the resources available.

Based upon evaluations of two previous courses on medical informatics and this combined course, we are developing a certificate program on health informatics at UPCH, recognizing the need for inter-institutional collaboration with well-established health informatics training programs. It is hoped that collaborations can be further extended to neighboring countries in the future.

## Competing interests

The author(s) declare that they have no competing interests.

## Authors' contributions

WHC collected the data, and led the analysis, interpretation, and manuscript drafting. JRH collected the data, participated in the data analysis and helped draft the manuscript. FMW made substantial contributions to the data analysis and interpretation of data. ACL, PJG, MZ, SF, AMK and KKH participated in planning the course, reviewed the manuscript and made significant comments. All authors read and approved the final manuscript.

## Pre-publication history

The pre-publication history for this paper can be accessed here:



## Supplementary Material

Additional file 1 Questionnaires (MI and BIO). Pre/post test questionnaires for both tracks (Medical Informatics and Bioinformatics). Click here for file
