# Supplementary material for: Evaluation of a joint Bioinformatics and Medical Informatics international course in Peru
Source: BMC Med Educ. 2008 Jan 14;8:1. doi: 10.1186/1472-6920-8-1 (PMC2265703; doi:10.1186/1472-6920-8-1)
Supplement: Additional file 1 — Questionnaires (MI and BIO). Pre/post test questionnaires for both tracks (Medical Informatics and Bioinformatics). [file 1472-6920-8-1-S1.pdf]

Copyright of this questionnaire belongs to Universidad Peruana Cayetano Heredia (Peru) and the University of Washington (Seattle).
© 2005. Universidad Peruana Cayetano Heredia and the University of Washington.
Contact wcurioso@u.washington.edu for permission

Appendix 1

PRE/POST TEST QUESTIONNAIRE
MEDICAL INFORMATICS

English

1. Which of these is not one of the core requirements for an organization working to *gain* commitment to large-scale change?
a) Developing a robust technology infrastructure supporting change
b) Ensuring organizational readiness for change
c) Communicating a clear vision of the proposed change
d) Promoting participation in the change effort

2. Which are the advantages of using controlled (indexing) vocabulary in searching?
a) Avoids problems of synonyms, spelling and other word variants
b) Groups similar concepts together regardless of actual words used
c) Enables use of "familial" relationships
d) All of above

3. Part 11 of the Code of Federal Regulations (CFR) of the U.S. Food and Drug Administration (FDA) states:
a) How develop and validate clinical software
b) Norms on the developing and validating clinical software
c) Some concepts on how to develop and validate clinical software
d) NA

4. What attributes of a surveillance system should be measured?
a) Simplicity
b) Specificity
c) Stability
d) a and c.
e) b and c.

5.What is the first thing needed in establishing a surveillance system?
a) Justification
b) Cooperation
c) Case Definition

6. When a patient is asked by hospital administrative staff specifics about his or her medial status, this is a violation of which:
a) Privacy
b) Confidentiality
c) Security

Copyright of this questionnaire belongs to Universidad Peruana Cayetano Heredia (Peru) and the University of Washington (Seattle).
© 2005. Universidad Peruana Cayetano Heredia and the University of Washington.
Contact wcurioso@u.washington.edu for permission

7. The files piled up on a nurses desk slip on the floor and skatter everywhere. A person passing by helps pick them up and sees personal medical information of someone he knows in the files. What type of violation is this?
a) privacy
b) confidentiality
c) security

8. An electronic medical record is:
a) A clinical data repository
b) A system to enter clinical data
c) A set of registration and transaction systems and clinical reports.
d) NA

9. What is a qualitative measure of the effects of handhelds on bacteriology result tracking?
a) The percent change in entry errors
b) The feedback from the users
c) Memory size of the handheld

10. What are quantitative measures of the effects of handhelds on bacteriology result tracking?
a) The percent change in entry errors
b) The feedback from the users
c) Memory size of the handheld

11.Which of the following causes the most resistance from people in an organization?
a) technological change
b) organizational change
c) social change

12. Successful leadership of large-scale informatics change in healthcare organizations require all but which of these characteristics?
a) Political skills
b) Analytical skills
c) Management skills
d) Technical skills

13 Validating software means:
a) Confirm that the user needs were accomplish
b) Check the internal validity
c) Evaluate the performance of the software
d) NA

Copyright of this questionnaire belongs to Universidad Peruana Cayetano Heredia (Peru) and the University of Washington (Seattle).
© 2005. Universidad Peruana Cayetano Heredia and the University of Washington.
Contact wcurioso@u.washington.edu for permission

14. What are the reasons for an evaluation study of handhelds use?
a) Measure the capacity of the handhelds
b) Measure the impact of the handhelds on the work pattern of the users
c) Measure the work load of the users

15. Indicate which of the following activities does not employ Free/Open Source Software?
a) Research in Basic and Applied Science
b) Government and Administration
c) Building of commercial software
d) Production of computer animated movies
e) Control systems in vehicles

16. In your understanding, what is the main characteristic of Free/Open Source Software?
a) The software can be downloaded and used with no need for payment.
b) The software gives you access to the source code, allows changes, and legal redistribution
c) The software belongs to nobody; they do not have a copyright and thus are in the public domain
d) They are software that allows you to see their source code, under certain rules given by the provider, and that cannot be changed or redistributed.

17. Which one is not an example of a distributed system?
a) Electronic mail
b) Banking transactions
c) On-line games (ie. Everquest, Final Fantasy XI, etc.)
d) Listening to a music CD on a computer
e) Downloading files from a web site

18. In which of the following databases you will find data of the sequence of the *Leishmania infantum* genome?:

a) Pubmed
b) Genbank/Genome
c) GeneDB
d) PlasmoDB
e) All of them

19. Which of the following is NOT a difference between licensed content and open access?
a) Licensed content is restricted to authenticated users only.
b) Licensed content is leased, not owned.
c) Open access content can increase the exposure of research findings.
d) Licensed content is of higher quality than open access.

Copyright of this questionnaire belongs to Universidad Peruana Cayetano Heredia (Peru) and the University of Washington (Seattle).
© 2005. Universidad Peruana Cayetano Heredia and the University of Washington.
Contact wcurioso@u.washington.edu for permission

20. Why use genomics?

a) Shows differences between species or strains.

b) Gives a compartmental view of the organism.

c) Identifies the energy potential of the organism.

Copyright of this questionnaire belongs to Universidad Peruana Cayetano Heredia (Peru) and the University of Washington (Seattle).
© 2005. Universidad Peruana Cayetano Heredia and the University of Washington.
Contact wcurioso@u.washington.edu for permission

PRE/POST TEST QUESTIONNAIRE BIOINFORMATICS

English version

1. When a patient is asked by hospital administrative staff specifics about his or her medial status, this is a violation of which:
a) Privacy
b) Confidentiality
c) Security

2. The files piled up on a nurses desk slip on the floor and skatter everywhere. A person passing by helps pick them up and sees personal medical information of someone he knows in the files. What type of violaton is this?
a) privacy
b) confidentiality
c) security

3. Sequence data from the *Leishmania infantum* genome projects can be found in which of the following databases?
a. Pubmed
b. Genbank/Genome
c. GeneDB
d. PlasmoDB
e. All of the above

4. Which of the following uses an intrinsic approach to gene prediction/annotation?
a. Blastn
b. Testcode
c. GlimmerM
d. Blastp
e. b and c

5. MUMmer is an algorithm designed to:
a. Assemble sequence reads into consensus sequence
b. Compare genome sequences
c. Predict genes in genomic sequence
d. Perform multiple sequence alignment
e. Analyze microarray data

Copyright of this questionnaire belongs to Universidad Peruana Cayetano Heredia (Peru) and the University of Washington (Seattle).
© 2005. Universidad Peruana Cayetano Heredia and the University of Washington.
Contact wcurioso@u.washington.edu for permission

6. The effectiveness of microarray analyses is limited by:
a. Inappropriate experimental design
b. Incorrect statistical analysis
c. Incorrect gene annotation
d. all of the above
e. a and c

7. For which of the following is overrepresentation used for?
a. One species and one gene
b. Multiple species and one gene
c. Multiple genes and one species
d. Multiple genes and multiple species

8. What kind of complications can arise from sequencing genomes?
a. Multiple identical copies of the genome
b. Repeats in the DNA
c. Breaking the genome into small fragments in random places.

9. Which of the following is one of the 9 future challenges for Bioinformatics?
a. Precise modeling of DNA splicing
b. Development of gene revolution
c. Precise model of transcription initiation and termination

10. Which of the following is true
a) The availability of the *Treponema pallidum* genome sequence has facilitated the identification of nutrients required for the cultivation of T. pallidum in vitro
b) The T. pallidum genome has allowed the development of a highly effective vaccine.
c) The genome sequence has shown that this spirochete is genetically very similar to oral and intestinal treponemes
d) The genome sequence has allowed the identification of mechanisms of immune evasion
e) All of the above.

11. Mutation mapping can be used to identify:
a) Inserts
b) Deletions
c) Copy Number Differences
d) Breakpoint analysis
e) All of the above

12. What type of sample is hybridized to the resequencing array?
a) DNA
b) RNA
c) Protein
d) DNA/Protein complex
e) All of the above

Copyright of this questionnaire belongs to Universidad Peruana Cayetano Heredia (Peru) and the University of Washington (Seattle).
© 2005. Universidad Peruana Cayetano Heredia and the University of Washington.
Contact wcurioso@u.washington.edu for permission

13. In the ChIP-Chip assay the antibody is used to precipitate what?
a) DNA
b) RNA
c) Protein
d) DNA/DNA Complex
e) DNA/Protein Complex

14. Chip-Chip assays can be used for all of the following except:
a) Gene Discovery
b) Promoter Discovery
c) Chromatin Structure Analysis
d) Mutation Mapping
e) Regulatory Control of Transcription

15. In the CGH application what are the 2 types of samples which are compared?
a) Genomic DNA & Protein
b) Genomic DNA & Genomic DNA
c) Genomic DNA & Cytosolic RNA
d) Cytosolic RNA & Protein

16. You applied for a researcher position at PharmaPeru and during the site visit they confront you with their purine nucleoside phosphorylase inhibitor development program. They show you 3 inhibitors with known binding mode and IC50 values (A, B, C), and ask you if they should bother to synthesize a related 4th one (D) to improve the affinity. (Assume that the IC50s are dissociation constants.)

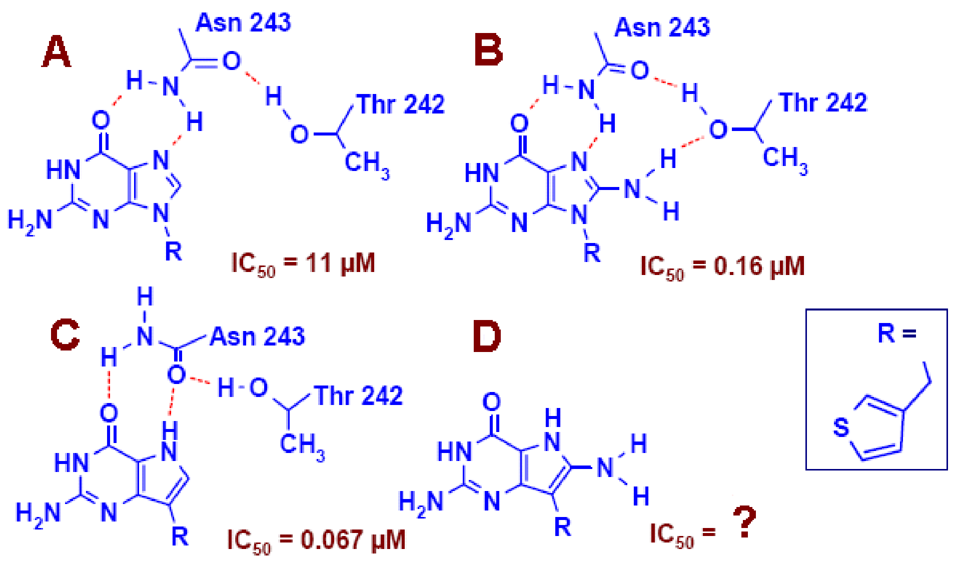

Copyright of this questionnaire belongs to Universidad Peruana Cayetano Heredia (Peru) and the University of Washington (Seattle).
© 2005. Universidad Peruana Cayetano Heredia and the University of Washington.
Contact wcurioso@u.washington.edu for permission

a. D will be as potent as C
b. D will be about 70-fold more potent than C
c. D will be about 1000-fold less potent than C
d. It is impossible to predict.
e. D will be as potent as B

17. The PharmaPeru group keeps probing your knowledge about molecular interactions.

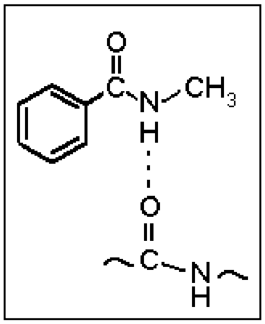

This time they prefer not to reveal the identity of their drug target. Explain the difference in binding constants between 3 ligands binding to protein Z assuming that there is no significant change in binding mode. Ligand 1, N-methyl-benzamide makes a hydrogen bond to protein Z. Ligand 2 = methylbenzoate, and ligand 3 = ethylphenylketone. The Kd's are 0.76, 660 and 1.40 □M respectively.

Please, consider that that a Kd refers to an equilibrium. This seemingly easy question may require a full page of analysis if you do a decent job!

a. It is just a matter of the strength of H-bonds, with making the strongest ones, then esters, then ketones.
b. Ligands 1 and 3 are rigid, while ligand 2 shows flexibility around the ester bond. Therefore, ligands 1 and 3 are about equally strong, and 2 is almost 1000-fold weaker.
c.There is no rational explanation.
d. It is a balance between hydrogen bond formation and solvation. Amides form strong H-bonds, esters decent ones, ketones yet weaker ones. Also we need to take into consideration the nature of the receptor. There is a cost to desolvate the amide and the ester, an little cost to desolvate the ketone.
e. It is a balance between hydrogen bond formation and solvation. Amides and esters form strong H-bonds, ketones not. There is no cost to desolvate the amide, a large penalty to desolvate the ester, an little cost to desolvate the ketone.

18. The easiest way to gain a lot of affinity of a ligand in drug design is:
a. To introduce a double bond to reduce the conformational entropy.
b. To introduce a salt bridge as Coulomb's law says that the energy of interactions between opposite charges is very large.
c. To add a hydrophobic substituent to gain affinity by the hydrophobic effect.
d. To make a symmetrical molecule to reduce the rotational entropy upon binding.
e. To introduce a new hydrogen bond.

Copyright of this questionnaire belongs to Universidad Peruana Cayetano Heredia (Peru) and the University of Washington (Seattle).
© 2005. Universidad Peruana Cayetano Heredia and the University of Washington.
Contact wcurioso@u.washington.edu for permission

19. Researchers discovered 2 inhibitors of the matrix protease stromelysin, labeled A (Kd= 17 mM) and B (Kd=20μM). Subsequently, they linked A and B together by a methylene linker and obtained C. Predict the Kd of C. Assume that it costs 0.5 kcal/mol to freeze out a rotatable bond, and that the entropy loss for immobilizing a rigid molecule is 4 kcal/mol.

```
      H                          C=C     C=C
      |                         /   \   /   \
HO-N-C-CH3 (A)             HO-C     C- C     C  (B)
        ||                      \\  //  \\  //
        O                        C-C     C-C
                                            \
                                             CN
```

```
       H                          C=C     C=C
       |                         /   \   /   \
HO-N-C-CH2-CH2-O-C     C- C      C       (C)
       ||                        \\  //  \\  //
       O                          C-C     C-C
                                            \
                                             CN
```

a. 0.016 nM
b. 630 nM
c. 5 nM
d. 0.05 nM
e. 27 nM

20. Often X-ray crystallographers observe well ordered water molecules in protein binding sites. Drug designers have to decide whether to replace a water molecule with part of the newly designed ligand or to leave it in and have it mediate interactions between the protein and the ligand. Which of the following considerations about this dilemma is true and relevant?
a. Well ordered waters always contribute favorably to binding enthalpy and should be left in place.
b. Waters can make H-bonds and therefore should always improve the free energy of binding of a ligand if mediating H-bonds can be made.
c. Waters should always be displaced because displacing them costs no energy. They either form H-bonds in the binding site or they form them in bulk water.
d. Displacing waters is always entropically favorable.
e. Waters in crystal structures are artifacts of crystallization. They should be ignored.
